# A Comparison of Checklist and Domain-Based Ratings in the Assessment of Objective Structured Clinical Examination (OSCE) Performance

**DOI:** 10.7759/cureus.40220

**Published:** 2023-06-10

**Authors:** Ahmed Mahmoud

**Affiliations:** 1 Family Medicine and Polyclinics, King Faisal Specialist Hospital and Research Centre, Riyadh, SAU

**Keywords:** global rating scales, ratings, domain-based, checklist, osce, assessment

## Abstract

Introduction: The Objective Structured Clinical Examination (OSCE) is the mainstay of clinical assessment in the final-year undergraduate Family Medicine clerkship at King Faisal Specialist Hospital and Research Centre (KFSHRC). The gold standard for OSCE assessment is the checklist rating, completed by physician examiners. Numerous studies have suggested that global or domain-based OSCE ratings may be a better indicator of competence than checklist ratings. The aim of this study was to examine the utility of domain-based OSCE ratings in the context of final-year, undergraduate, Family Medicine OSCE examinations in Riyadh, Saudi Arabia. This is akin to an exercise in quality improvement, as we continuously look for ways to improve our OSCE assessment processes.

Methods: This study utilised a quantitative methodology. Three final year OSCE exams were chosen. Physicians rated each student using a checklist score and using a more holistic domain-based score. Physician checklist scores and physician domain-based scores were then compared, and correlation was assessed. We also looked at the internal consistency of the scoring methods.

Results: A significant correlation was found between checklist and domain-based scores by physicians for all exams (r=0.858, p<0.01), with a good internal consistency for these methodologies for all exams.

Conclusion: The results demonstrate that both checklist and domain-based scores offer some benefit to the assessment, with a similar internal consistency and strong correlation. Domain-based ratings should be utilised for softer skills that are not easily assessed by checklists. There is clearly a need to rethink our OSCE assessment. The assessment should combine checklist and domain-based physician scores. As trainees become more experienced, checklist OSCE may penalise directness and efficiency, while domain-based ratings would offer a better appraisal of competence, and have been shown to be more sensitive to the level of training and expertise. Changing the assessment methods will lead to necessary changes in the student approach to the OSCE and improve authenticity and validity.

## Introduction

The need for fair, reliable, and accurate assessment of medical trainees is a challenge that has been studied extensively. Everyone recognises, for example, that a good family physician is someone whom patients would happily be treated by, and be comfortable following up with regularly. The challenge is to design assessments that can measure these qualities that are difficult to define, and even harder to assess. Summative assessment, or assessment of learning, allows us to grade performance to ensure that the trainee has reached the required standard to practice in a safe and competent manner, as well as to provide a way of discriminating among levels of competence as trainees compete for higher training posts [1,2].

The Objective Structured Clinical Examination (OSCE) is a standardised simulation intended to be carefully designed in alignment with learning outcomes that are mapped to curriculum objectives [3]. However, some authors suggest that the OSCE may fail to measure unique skills and behaviours that reflect the reality of good medical practice as not only a science, but also an art [4]. Trainee attitude, communication, empathy, professionalism and teamwork are just some of those domains that are notoriously difficult to assess by OSCE evaluation. O’Sullivan and Toohey looked at a number of studies in Australia and concluded that a higher proportion of iatrogenic injury in Australian hospitals was related to lapses in professionalism than to deficiencies in knowledge, and they went on to argue that students with well-developed professionalism would be significantly less likely to be involved in situations linked to a medical error, and even if involved, they would be more capable of dealing with the error and its repercussions in an open and effective manner [5]. These findings help reinforce the importance of building and appropriately assessing these crucial ‘softer’ skills.

The OSCE has long been championed as the benchmark for clinical assessment, with detailed binary checklists containing clear, pre-determined criteria for recording the presence or absence of specified behaviours. These might produce more reliable, valid, objective and therefore unbiased results than the global assessment of long case or overall clerkship performance [6]. The purpose of the OSCE is to simulate realistic problems or scenarios encountered in the clinic. It was expected that the OSCE’s authentic simulation of problems or scenarios encountered in the clinic would allow the trainee to be immersed in a standardised, objective environment [7,8]. It was hoped that the structured nature of the OSCE with a checklist of tasks, observed by an expert, would be more resistant to bias, with explicit, unambiguous expectations, and examiners acting as impartial observers, removing, or at least significantly reducing subjectivity and the need for examiner interpretation [9,10].

However, Norman and Feightner opined that the essence of the effective physician was not merely knowing how best to perform what they are asked to perform, but actually determining what it is that needs to be done and focusing on the most essential issues [11]. As such, the checklist OSCE is not rewarding true competence and a holistic approach to problems, but instead encouraging a discrete, rote and scattergun approach to consultations. In more recent times, the checklist OSCE has come under criticism for rewarding the detailed linear accumulation of facts, rather than more focused and efficient data gathering, while generally neglecting empathy, consultation structure and organisation of knowledge, and thus failing to capture true clinical competence and higher levels of expertise [12,13].

Dreyfus and Dreyfus have described five stages of development in trainees, starting at the novice level, where large amounts of data are collected in the consultation, in a non-structured approach, in an attempt to formulate a diagnosis [14]. The trainee then develops through the various stages that culminate at the expert level, where the practitioner would take a much more focused and structured approach to information gathering, responding to prompts and observations, leading to much more rapid diagnosis and improved efficiency. Expert clinicians skip less relevant steps, focusing on the crux of the issue, while maintaining excellent quality of care. Checklists would therefore penalise experts unjustly for being more direct, focused, effective and efficient [15]. In addition, the ability of checklists to adequately capture complex human emotions and behaviours such as empathy, organisation of ideas and professionalism is questionable [16].

Domain-based ratings are more holistic judgements of performance, not confined to specific behaviours or actions that must be performed. As a result, they are much less likely to encourage rote memorisation of knowledge or behaviours. Domain-based ratings therefore allow for the fact that the same problem can be approached in different ways, allowing for the recognition of superior levels of competence and efficiency, geared more towards quality than quantity and breadth of coverage [10]. In essence, the opportunity for experts in their field to make subjective holistic judgements is lost with checklists.

Van der Vleuten found that as a predictor of future performance levels, domain-based ratings demonstrated significantly enhanced predictive power when compared to checklist ratings, while by contrast, checklists did not appear to provide a true measure of overall professional competence [17].

Several metrics are now commonly used for analysing the reliability of an OSCE assessment. Cronbach’s alpha is a measure of the internal consistency of a test, with a minimum alpha value of 0.7 or 0.8, suggesting a reliable test [3]. However, an alpha score above 0.9 would indicate redundancy, while a low alpha does not necessarily signify poor design, and may be due to significant differences in what the stations are measuring. OSCE stations measuring a variety of concepts or knowledge areas might be expected to produce a low Cronbach’s alpha score. Thus, we may be confusing homogeneity with internal consistency [18]. Another common metric used to assess station reliability is the correlation between the expert domain-based ratings and the expert checklist scores, with a correlation of r>0.7, or r>0.8 indicating a reliable station [19].

Ward et al. showed that the addition of the checklist scores to the domain-based ratings did not appear to significantly affect the overall OSCE reliability in a positive or negative way [20]. This would certainly seem to make sense, bearing in mind that medical decision-making, and thus competence, is based on holistic appraisal of all the available information in complex ways. This appraisal would seem to be best performed by experts in the field who understand this process, and might be very difficult to reduce to a tick-box formula.

Clearly, it is important that the OSCE reflects the reality of clinical consultations as much as possible, and that the assessment process is valid, i.e., it truly measures the important knowledge, skills and attitudes that are required for the competent practice of medicine. OSCE has become the gold standard of undergraduate medical assessment, and it has been assumed that OSCE performance can be readily extrapolated to performance in real clinical practice [21]. Any disconnect between OSCE scenarios and reality may lead to the trainee adopting one approach in the OSCE, while behaving in a completely different manner in real consultations. Trainees may alter their performance to tick the checklist boxes and demonstrate the required competencies. This raises the question of whether purely checklist-based OSCE performance can truly be generalised to real-world performance [4].

Although there has been considerable debate in the literature about the validity of checklists versus domain-based scoring, few studies have compared these two methods directly [22]. In an attempt to address this knowledge gap, members of the clinical faculty at Al-Faisal University in Riyadh looked at our own undergraduate family medicine OSCE to investigate the potential for changing the checklist scoring standard, replacing it with domain-based ratings or combining the two methodologies.

## Materials and methods

Sampling strategy

Undergraduates each complete three 10-minute OSCE stations with simulated patients (SPs) and three different physician examiners at the end of their fifth year Family Medicine clerkship. The study population comprised final year students undergoing this OSCE between October and December 2020. Three groups of students were included, with approximately 20-25 students per exam, for a total study population of 71 students, passing through a total of 213 OSCE stations. Sampling strategy was purposive non-random sampling, as every student who passed through the Family Medicine clerkship at King Faisal Hospital during the named period was included.

Scoring

In each of the OSCE stations, one physician examiner scored the OSCE using a standard checklist rating method, as well as domain-based ratings organised into four domains: clinical competence, communication skills, knowledge and empathy. An example of a checklist rating scoresheet (out of 30) and a domain rating scoresheet (out of 20) is provided in Appendix 1 and Appendix 2, respectively. Examiners were purely silent observers and evaluators of the consultation during the OSCE, neither asking questions nor giving prompts. The examiners were instructed to add up the total checklist scores only after they had entered the domain-based scores, to reduce the likelihood that the checklist scores might affect the domain-based scores. A rubric was prepared for examiners to standardise the marking process (Appendix 3). The rubric was used during the training sessions to practice some scenarios with the participants and to resolve any inconsistencies or confusion in rating trainees. Students were aware that the official documentation of their summative performance would be from checklists, as has been the practice at this institution since the clerkship began nine years ago. From the student’s perspective, nothing about the assessment environment was different from normal.

Data analysis

The analysis of the quantitative data started with descriptive statistics including range, mean, standard deviation, skew and kurtosis for each of the scoring methods. Next, correlation was checked between the two data sets, examiner checklist ratings and examiner domain-based ratings. Physician domain ratings for trainees were correlated with physician checklist ratings for those same trainees in the same OSCE stations. Correlation was performed using the Spearman's rank correlation, a non-parametric test, because the data was not normally distributed. Interstation reliability was also checked using Cronbach’s alpha, for each scoring method. Statistical analysis was performed with SPSS, version 27 (IBM Corp., Armonk, NY).

The study was approved by the University of Dundee School of Medicine Research Ethics Committee (SMED REC number 20/105) as well as the Research Advisory Committee at King Faisal Specialist Hospital (RAC#2201125).

## Results

Quantitative data

**Figure 1:**
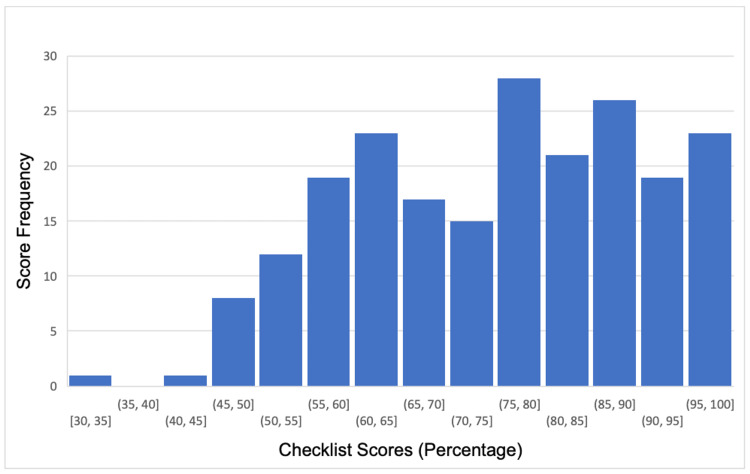
Distribution of physician checklist scores

All 71 eligible Al-Faisal University final-year medical students agreed to participate after informed verbal consent was obtained. In OSCE round 1, 20 students were recruited, in round 2, 25 students were recruited, and in round 3, 26 students were recruited. No students declined or withdrew from the study. Figure 1 shows the distribution of the checklist total scores over the three OSCE rounds. All scores were converted into percentages.

**Figure 2:**
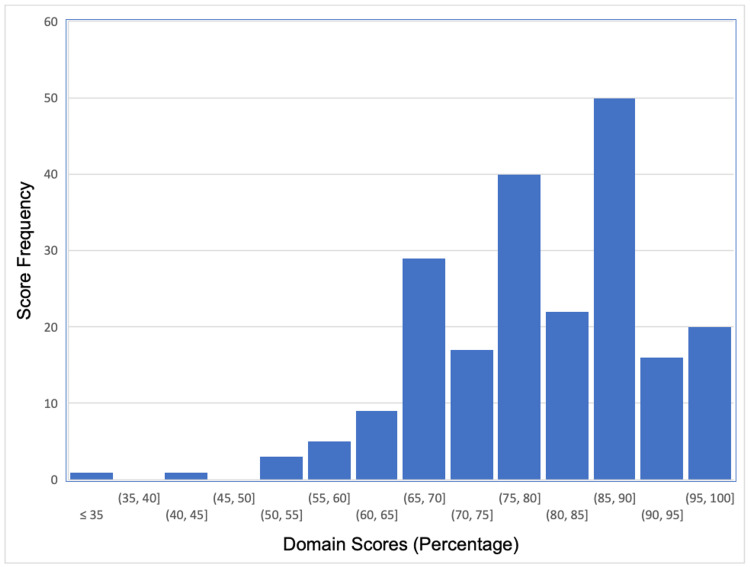
Distribution of physician domain-based scores

Figure 2 shows the same distribution for domain-based total scores. The histograms suggest that the OSCE scores were not normally distributed, whichever scoring method was utilised.

This observation was confirmed by the Shapiro-Wilk test: p values were <0.05 for every station in all three rounds. The distribution of checklist scores had a skew of -0.73 and a kurtosis of 0.86, while the distribution of domain-based scores had a skew of -0.28, and a kurtosis of -0.77. This indicates that the checklist scores were moderately negatively skewed, while the domain-based scores were only slightly negatively skewed. Regarding kurtosis, checklist scores demonstrated a positive kurtosis suggesting a more peaked distribution, whereas domain-based scores demonstrated a negative kurtosis, suggesting a flatter distribution with thinner tails.

Table 1 describes the quantitative data that was gathered from the OSCEs. The table illustrates the maximum and minimum total scores obtained by any student in each method (checklist and domain) and in each specific domain in the case of physician domain ratings. It also shows the average rating obtained by students by each method and in each domain. The data includes the 71 students from all three cohorts.

**Table 1:** Mean and range of scores across various ratings Comms, communication skills

Rating	Maximum student score (%)	Minimum student score (%)	Mean student score (%)
Physician checklist total	100	33.3	80.5
Physician domain total	100	30	77
Physician domain Comms	100	20	76.5
Physician domain Knowledge	100	40	80.8
Physician domain Empathy	100	20	73.7
Physician domain Competence	100	40	77.2

Both checklist and domain-based scoring produced some scores of 100%, i.e. maximum scores were given to some students with either method. Minimum scores were similar with the two methods: 10/30 (33%) with checklists and 6/20 (30%) with domains.

Table 2 shows Cronbach’s alpha scores for each of the rating methodologies for each of the cohorts. It represents the internal consistency of this scoring methodology across the OSCE stations for each of the different ratings methods, in each OSCE cohort. A high Cronbach’s alpha would suggest that students score consistently across all the stations of their OSCE. A low score would suggest the opposite, with inconsistent scoring, which might imply a problem with the entire exam, one of the stations, or the scoring methodology. Alternatively, it might simply mean that the assessment is multi-dimensional, measuring various, largely unrelated, skills and traits.

**Table 2:** Cronbach’s alpha for physician checklist and domain scores in each OSCE group OSCE, Objective Structured Clinical Examination

Variable	Cohort 1	Cohort 2	Cohort 3
Physician checklist total	0.795	0.428	0.758
Physician domain total	0.765	0.461	0.729

Table 3 illustrates the Spearman rank correlation between the two scoring methods. Total checklist scores for each cohort of students were correlated against total domain scores. The correlations were then run again for the entire study population of 71 students.

**Table 3:** Spearman's rank correlation coefficients for highlighted correlations All Spearman rank correlations were significant at the p<0.01 level.

Correlation	Cohort 1	Cohort 2	Cohort 3	All cohorts
Physician checklist vs. physician domain total	0.832	0.886	0.868	0.858

There was a strong correlation between domain-based total and checklist total scores for the entire group, with a correlation coefficient of 0.858 (p<0.01).

## Discussion

The results suggest that the correlation between physician checklists and domain-based scores is strong and positive. Cronbach’s alpha comparisons for these two grading methods also show similar levels of internal consistency for both physician checklist and domain-based scores for each of the three separate OSCE examinations. This is in agreement with previous studies, which found similar reliability and a good correlation between the two scoring methods [23,24]. One notable finding was that Cronbach’s alpha was significantly lower for the second OSCE cohort, indicating a greater variation (reduced consistency) between the scores at each of the OSCE stations in the second cohort. This may have been due to the fact that the skills being tested were more heterogeneous in the second OSCE, or there may be problem with one or more of the stations.

Assessment drives learning. Clearly, students would be aware of the system of assessment and would adapt their behaviours and consultation manner to suit the perceived system of evaluation. A checklist evaluation will prompt a more thorough approach to the consultation, whereas a domain-based scoring system might prompt a more focused and patient-centred consultation. McIlroy et al. found that when students were expecting a checklist evaluation, they performed better on the checklist scoring system and worse on the domain-based evaluation [25]. However, the opposite was also true, such that the students who were expecting a domain-based rating performed significantly better in the domain scores than the checklist method. Trainees who anticipated the checklist evaluation asked multiple closed questions focused on information gathering, whereas those anticipating domain-based scores asked far more open questions, paying particular attention to cultivating relationships with the SPs.

Experienced, knowledgeable physicians do not solve real-life problems in a purely ‘checklist’ manner. At the very least, it appears that a mix of assessment indicators is required to enrich the process and lead to the construction of more holistic, personalized assessment information that could be used in both a formative and a summative capacity to improve patient care.

In a recent study, Giemsa et al. found that domain-based ratings were more sensitive discriminators than checklist scores in differentiating between clinical and pre-clinical undergraduate medical students in a family medicine OSCE [26]. This was especially the case with regard to communication skills and empathy, as well as general clinical competence. Checklists are essentially a beginner’s approach to problem solving. Expert clinicians, like experts in other fields, have learned to approach problems holistically and to be selective and efficient, rather than necessarily thorough. An illustration of the discriminant validity issue this represents, for checklist-centred OSCEs, was provided by Hodges et al., who found that such assessments could not differentiate adequately between family practice residents and board-certified family physicians [13]. In fact, they found that experienced physicians scored significantly lower than did residents and clinical clerks when rated on checklists, but significantly higher when rated on domain-based scores. Of course, there are also issues with domain-based ratings, including the fact that they are clearly more subjective, more difficult to standardise, and more dependent on physician knowledge and experience, in addition to the fact that it would be harder to defend the grade given by the domain-based scoring method, if it were challenged in a high-stakes assessment.

The most important aspect of the evaluation is the OSCE station design itself. Station designers should ask themselves whether the stations are testing what they need to test, with the correct methods and suitably trained evaluators? Pell et al. found that ‘chunking’ or grouping together related checklist items into ratings scales, for example, overall introduction or pharmacological management, significantly improved OSCE reliability, and would encourage trainees to gain a deeper understanding of the topic, rather than learning checklists [3]. At the very least, the checklist might be used to ensure that all evaluators are reminded of the more concrete, mechanistic aspects of the OSCE performance. The answer may be a less rigid assessment process, incorporating aspects from different assessment methods and utilizing different assessors in well-structured, clinical scenarios, properly reflecting the curriculum blueprint.

Checklists might measure information-gathering and completeness more effectively, while domain-based ratings may be more suited to the appraisal of communication skills, empathy, consultation structure and general competence. Adding checklist items that pertain to these latter skills seems inappropriate, as the skills themselves transcend any individual consultation and are instead indicators of a holistic and efficient approach to patient care in general. The triangulation of all available assessment data allows us to paint a comprehensive picture of the trainee’s performance, which can be utilised in a formative or summative manner.

A possible limitation of this study includes its reliance on data from a single institution. This may mean that there is limited generalisability of findings. Additionally, examiners and stations were different for each examination. This was a logistical necessity. Another limitation is that the trainees were all final-year undergraduates. Trainees with different levels of experience, such as interns or residents, were not included in this study. Turner et al. found that the correlation in OSCE between domain-based scores and checklist scores increased for more advanced students in a physiotherapy department [27]. This may suggest that as trainees become more experienced and confident, they are able to combine acquired knowledge, technical skills, and more holistic skills, such as communication and organisational skills, emphasizing the importance of holistic assessment in these groups. Further to this, the training level of the examiners was variable, which might also affect their domain-based ratings. One would expect these ratings to become more reliable with increasing examiner expertise and experience.

Kane proposed a framework that provides an approach to evaluating the validity of an assessment [28]. This framework would suggest that the validity of the assessment of an OSCE performance depends on certain key steps, which include the ability to somehow translate performance into a score, then to extrapolate that score to the general performance environment and then finally to real life [29]. Extrapolating the score to the general performance environment and then to real life requires that the test is valid, meaning that the real-life environment is appropriately and adequately represented. This again emphasises the importance of an adequate number of expertly designed and peer-reviewed stations with blueprinting across the entire curriculum to ensure that the assessment is as fair a reflection of reality as can be reasonably constructed.

## Conclusions

The theme that seems to be emerging is that there is no ‘best’ way to undertake the OSCE examination. There are many variables that might affect one’s choice of assessment rating, including the level of the trainees, the domains being assessed and the resources available. Physician checklist and domain-based scores were reliably positively correlated, and both showed a reasonable internal consistency, with both rating methods having their place in undergraduate assessment.

Locally, there is certainly a need to improve the validity and reliability of our own OSCE assessments by increasing the number of stations, basing all the stations on real clinical scenarios to increase authenticity, and blueprinting the exam such that all aspects of medical expertise are tested, including knowledge, examination skills, communication skills, ethics, counselling, professionalism, advocacy and even teaching skills. Specific stations could be designed to test specific skills rather than trying to incorporate as many skills as possible into each station, which might lead to inadequate assessment of all skills.

A growing body of evidence supports the idea that domain-based ratings are more reliable than checklists, producing consistent, reproducible ratings, and providing a more accurate means of discriminating between different competence levels of trainees. Checklist items should be relevant, evidence-based, clinically discriminating items. Areas of competence that are not readily accessible to checklist scoring should be scored by domain-based ratings. Well-designed checklists, in association with domain-based ratings, have a vital role to play in the assessment of undergraduates, especially, as there is a process that novices need to go through to gather all the necessary information. Only by regularly going through this process will they eventually attain expert status, at which point a more focused consultation methodology would certainly have developed naturally in the physician. The transition from novice to expert involves a shift from analytic, piecemeal thinking to a more efficient holistic approach. As the trainees become more experienced, a shift to a more domain-based rating method would certainly seem to be warranted, to avoid punishing the efficiency that stems from experience and, indeed, expertise.

## Figures and Tables

**Figure 3:**
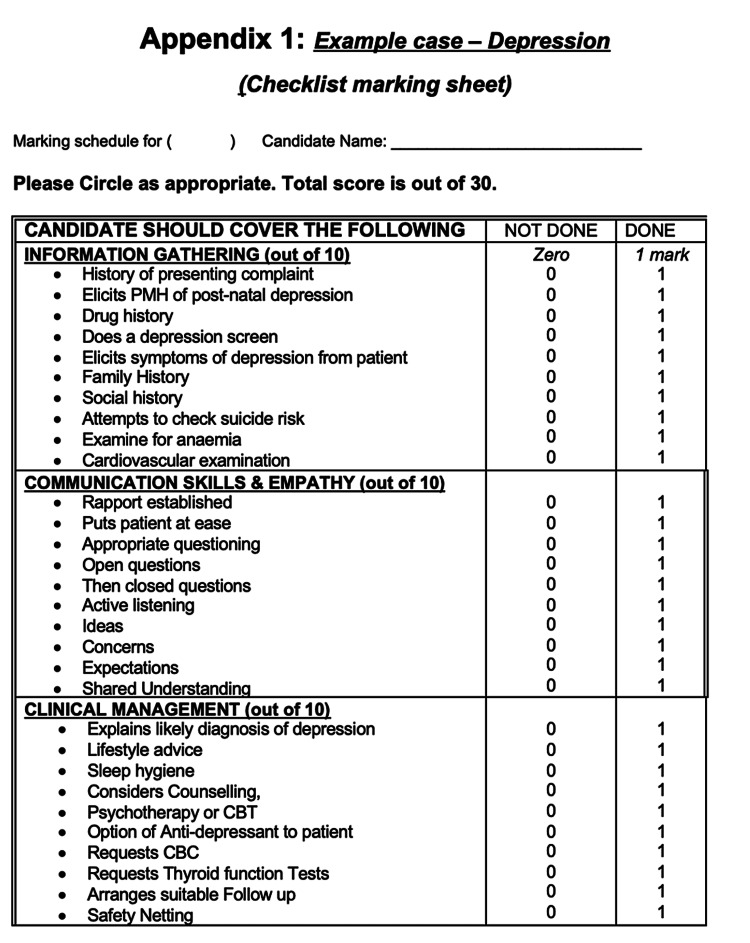
Example case PMH, past medical history; CBC, complete blood count; CBT, cognitive behavioral therapy

**Figure 4:**
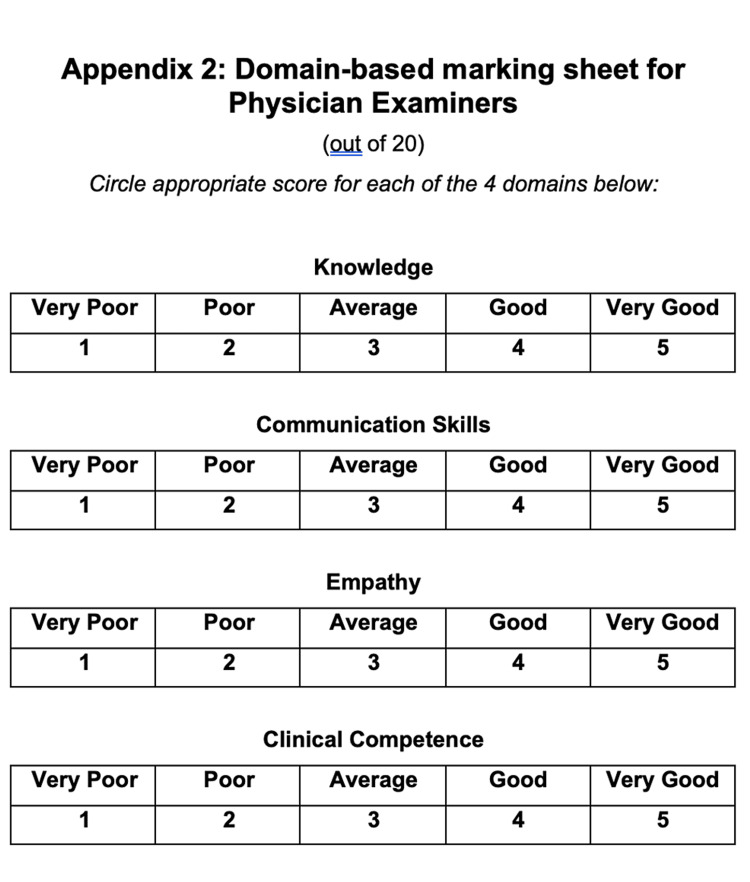
Domain-based example

## Appendices

Appendix 1

Appendix 2

Appendix 3 

**Table 4:** Rubric for examiners: How to assess students in OSCE domains OSCE, Objective Structured Clinical Examination

Domain	1 – Very poor	2 – Poor	3 - Average	4 – Good	5 – Very good
Empathy	Little or no empathy. No consideration to the needs of the patient. Self-involved, egocentric. No response to cues or concerns.	Understands that the patient has needs. Little response to patient needs. Limited perspectives. Brushes over patient concerns.	More connected to how the patient sees things and patient perspectives. Makes an attempt to address patient concerns.	Good connection to patient. Better attempt to address patient’s ideas, concerns and expectations. Patient feels his thoughts and feelings are being addressed.	Able to put himself in the patient’s shoes. Addresses patient’s ideas and concerns and actively responds. Patient feels completely at ease. Sympathetic.
Communication skills	Closed body language. Closed leading questions. Looking at paper or monitor. No eye contact. No rapport established. Patient not at ease. No information shared. Uses a lot of Medical jargon	Tense body language. Turns slightly to speaker. Minimal eye contact. Mostly closed questions. Concentrated on gathering info. Use of medical jargon	More relaxed. Intermittent eye contact. Turned more towards the patient. Some open questions. No significant response to patient concerns – passive listening.	Mirrors body language of patient. Sustained eye contact. Mixture of open and closed questions. Good rapport. Makes an attempt to share information with the patient. Minimal medical jargon.	Relaxed and open. Natural eye-contact. Listens attentively. Shows interest and respect. Starts with open questions and allows patient to express themselves. Active listening. Picks up cues. Puts patient at complete ease. Shares and checks understanding and management plan. No medical jargon.
Knowledge	Poor knowledge. No grasp of the subject matter. Gives inaccurate information to patient. Formulates no plan or inappropriate unrealistic plan.	Below average knowledge. Inadequate grasp of subject matter. Incomplete or incorrect plan. Little information given to patient.	Average knowledge. Reasonably accurate information. Formulates a reasonable plan. Could give more relevant information and more extensive plan.	Above average knowledge. Shows a good grasp of the subject matter. Offers the patient some relevant information and formulates a reasonable realistic plan.	Excellent knowledge. Knows the subject matter in depth. Gives relevant and accurate information and formulates fully appropriate and realistic plan.
Clinical competence	Student is incompetent, Disorganised consultation. Poor time management. Unable to prioritize. Unsafe. Unfit for internship.	Below average competence. Probably unfit for internship	Average competence. Student borderline for internship.	Above average competence. Student ready for internship.	Fully competent. Student has reached a high level. Organised consultation, professional, excellent time management, prioritizes well. Student well above the required level for internship.

Appendix 4

Participant Information Sheet - Physicians

You have been invited to take part in a research study. The following information will help you understand why the study is being done and what your role would entail. Please take time to read it and ask questions if you need any clarifications.

Who am I? I am Dr Ahmed Mahmoud, Family Medicine consultant at KFSHRC. The study will involve comparing different ratings methods for the OSCE exams and examining the usefulness of different ratings methods. As Physician examiners, your involvement would give us insights into new ways of assessing student performance.

What will taking part involve? As a Physician examiner, you would be evaluating the students as usual, using the checklist OSCE marking sheet. However, in addition to your usual checklist grade, you would be asked to grade each student in more general competency domains. The grading would be in 4 domains or aspects of the consultation. You would be grading the student’s knowledge, empathy, communication skills and competence. Grading would be on a 1-5 scale for each of these 4 domains. Prior to the OSCE exam, we will go through the grading sheets and how best to assess the students, with the help of a rubric which describes the key behaviours you might be looking for to aid you in assessment. As usual, only the checklist rating will count towards the student grade, while your domain-based ratings will not contribute to the overall grade and will therefore not affect the student in any way.

Do you have to take part? Participation is completely voluntary. You are free to refuse participation at any point up to the beginning of data analysis; if you prefer not to participate, you will only be required to grade the students with the OSCE checklist as usual.

For any further information: You can contact me, Dr Ahmed Mahmoud, at any time if you have any concerns or further inquiries. You will be given my pager number and hospital email address for this purpose.
